# Intracoronary administration of tirofiban during percutaneous coronary intervention facilitates patients with acute coronary syndrome

**DOI:** 10.18632/oncotarget.19179

**Published:** 2017-07-12

**Authors:** Helei Jia, Changqing Lu, Panli Sun

**Affiliations:** ^1^ Department of Emergency, Henan Province Hospital of Traditional Chinese Medicine, Zhengzhou, Henan Province 450002, China; ^2^ Department of Cardiology, Henan Provincial Hospital, Zhengzhou, Henan Province 450000, China

**Keywords:** percutaneous coronary intervention, acute coronary syndrome, randomized controlled trials, tirofiban

## Abstract

We assessed the efficacy and safety of tirofiban intracoronary versus intravenous administration during percutaneous coronary intervention for patients with acute coronary syndrome. The databases of PubMed, Web of Science, China National Knowledge Infrastructure, and WanFang Database were retrieved. A total of 437 articles were found, according to inclusive and exclusive criteria, 13 of which were finally included. Compared with subjects with intravenous administration, those with intracoronary administration were more likely to reach thrombolysis in myocardial infarction trial grade 3 flow (relative risk = 1.17, 95% confidence interval: 1.11–1.22), improve left ventricular ejection fraction (Standardized mean difference = 0.65, 95% confidence interval: 0.20–1.11). Intracoronary administration resulted in a reduced risk of major adverse cardiovascular events at 30-day follow-up (relative risk = 0.47, 95% confidence interval: 0.34–0.65). However, incidence of bleeding complications was not statistically significant between two groups (relative risk = 0.76, 95% confidence interval: 0.55–1.04). Intracoronary administration of tirofiban can be more effective in increasing coronary blood flow and microvascular perfusion, more effective in improving postoperative myocardial reperfusion, more significantly in reducing the incidence of adverse cardiovascular events at 30-day’s follow-up and improving the prognosis after percutaneous coronary intervention without increasing the risk of bleeding.

## INTRODUCTION

Acute coronary syndrome is a syndrome (set of signs and symptoms) due to decreased blood flow in the coronary arteries such that part of the heart muscle is unable to function properly or dies. Acute coronary syndrome is the primary cause of leading to death for cardiovascular patients [1]. Though this disease is less common than European and America population, recent statistics reports the increased trend. For patients with acute coronary syndrome, the primary treatment strategy was to restore occlusion of blood vessels using all different kinds of methods. Percutaneous coronary intervention (PCI) was a priority option [2]. However, expansion of balloon and stimulating effect of metal stents can cause endothelial injury, platelet activation and aggregation, adhesion, and lead to acute vascular occlusion and coronary artery embolism formation after PCI operative. Therefore, antiplatelet therapy was a very important part of PCI therapy [3].

Tirofiban is a receptor antagonist of GP IIb/IIIa, and its ability of antiplatelet has been confirmed by several randomized controlled trials [4, 5]. For patients with acute coronary syndrome who underwent percutaneous coronary intervention, there are two different ways of Tirofiban usage (intravenous vs intracoronary). Potential benefits and possible risks associated with intracoronary administration compared with intravenous were not fully understood. Studies from randomized controlled trials remained inconsistent. Previous meta-analysis was also underpowered to draw a determinate conclusion. Moreover, several studies with enough power have been published. Thus, we conducted a latest meta-analysis to assess the efficacy and safety of tirofiban intracoronary versus intravenous administration during percutaneous coronary intervention for patients with acute coronary syndrome. New evidence will provide important guidelines for clinical practice.

## RESULTS

### Study selection and general characteristics

**Figure 1:**
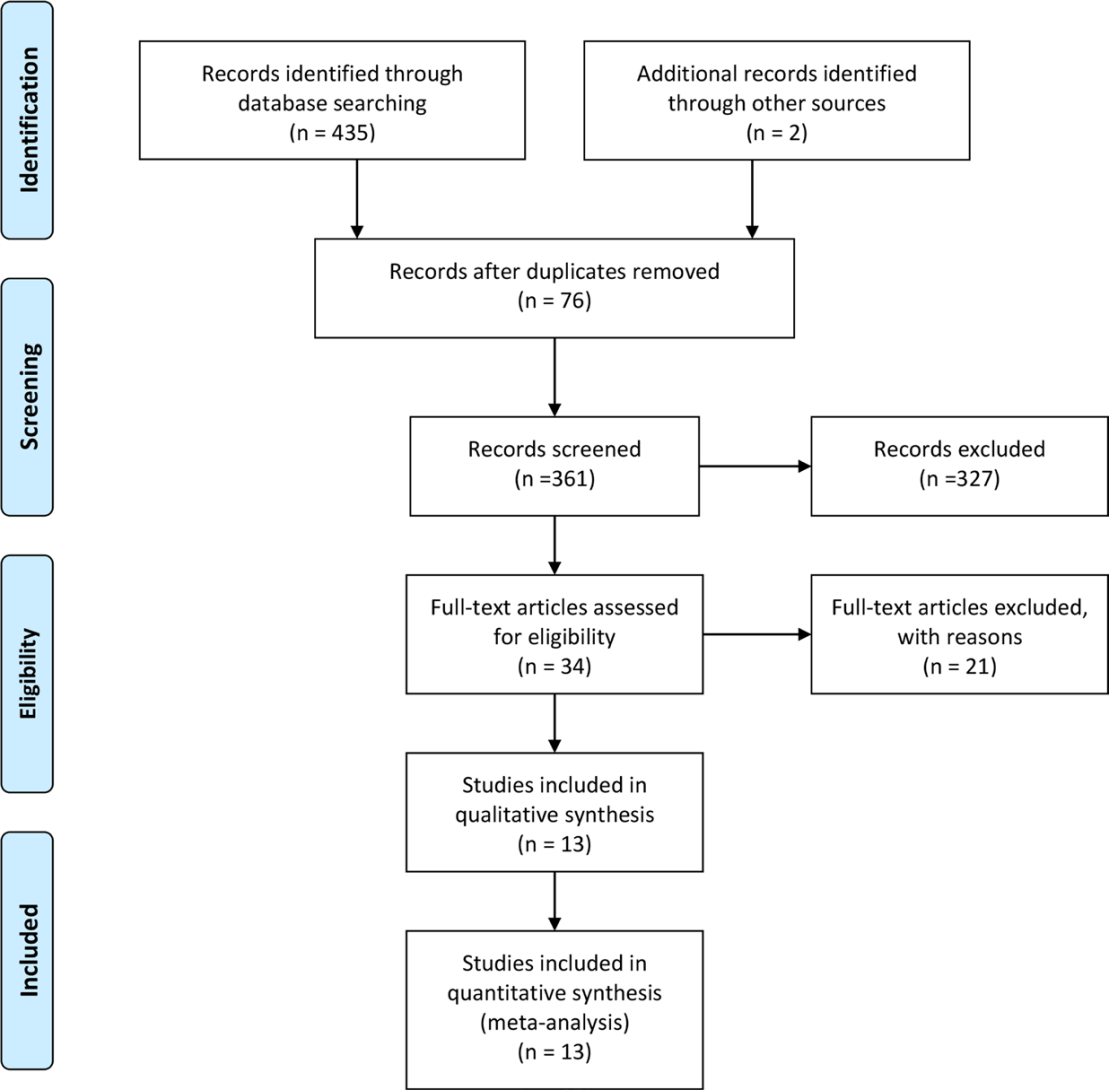
Selection of studies for meta-analysis

The selection flow of study was presented in Figure 1. Our initial search returned 435 records, and obtained 2 studies via other sources. After removing duplicates and screening the titles and abstracts, 34 studies were potentially eligible for inclusion. After reviewing the full-text, 13 studies finally entered the final meta-analysis. Some data were obtained by contacting with authors. Thirsty studies were published from 2008 to 2014 [6–18]. The total number of population was 1550. Most of study population were from Asian population, and the duration of follow-up were limited with 6 months. The sample size ranged from 52 to 453. All studies were conducted among adult population. Among the included trials, 13 studies reported the incidences of thrombolysis in myocardial infarction, 9 studies compared the left ventricular ejection fraction between intracoronary tirofiban and intravenous administration, 11 studies compared the cardiovascular adverse events occurrence between two ways, and 9 studies compared with bleeding complications incidences. The general characteristics of included studies were presented in Table 1.

**Table 1:** General characteristics of included studies in the meta-analysis

Author	Year	Mean age (T/C)	Duration of follow-up (m)	Sample size		outcomes	Tirofiban
				Trial group	Control group		
Wang, et al. [6]	2012	58.0/57.0	1	51	47	①②③④	10 ug/kg
He, et al. [7]	2012	-	1	31	21	①③④	10 ug/kg
Wang, et al. [8]	2013	66.3/68.0	1	91	91	①③④	5 ug/kg
Chen, et al. [9]	2013	62.1/61.3	1	44	46	①②③	10 ug/kg
Xue, et al. [10]	2013	63.8	1	55	53	①②③④	10 ug/kg
You, et al. [11]	2013	61.8/61.6	1	37	37	①②③④	10 ug/kg
Zhao, et al. [12]	2014	76.0	1.5	38	38	①②③④	10 ug/kg
Zhang, et al. [13]	2014	-	1	61	52	①②③	10 ug/kg
Wu, et al. [14]	2008	75.0	6	58	57	①②③④	10 ug/kg
Refik, et al. [15]	2010	55/56	1	36	48	①②	10 ug/kg
Candemir, et al. [16]	2012	69.4/70.9	1	34	22	①②③	10 ug/kg
Cevat, et al. [17]	2012	57/56	6	25	24	①②	10 ug/kg
Tian, et al. [18]	2013	64.7/64.6	6	229	224	①③④	10 ug/kg

T: trial group; C: control group; ①thrombolysis in myocardial infarction trial ②left ventricular ejection fraction ③cardiovascular adverse events ④bleeding complications.

### Assessment of quality

We used the Cochrane risk of bias tool to assess the risk of bias. Two investigators independently conducted this procedure. The Supplementary Figure 1 shown the details of risk bias. Three studies were classified as high risk of bias, five for unclear risk, and three for low risk bias. The random sequence generations were obtained in all studies. Blinding of outcome assessments was unclear or rarely reported in these studies.

### Pooled results

#### Thrombolysis in myocardial infarction trial

**Figure 2:**
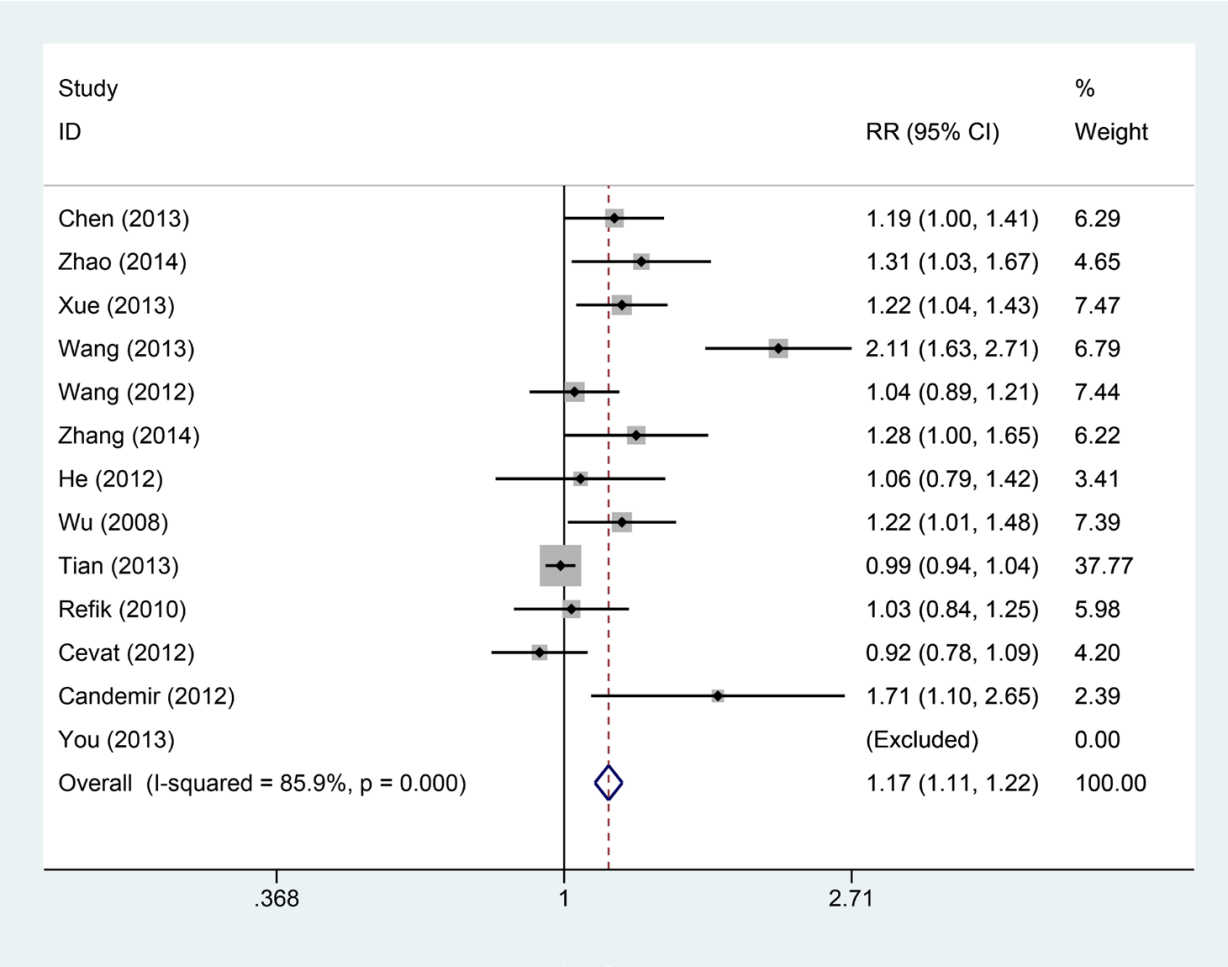
Comparisons of thrombolysis in myocardial infarction between intracoronary vs intravenous

**Figure 3:**
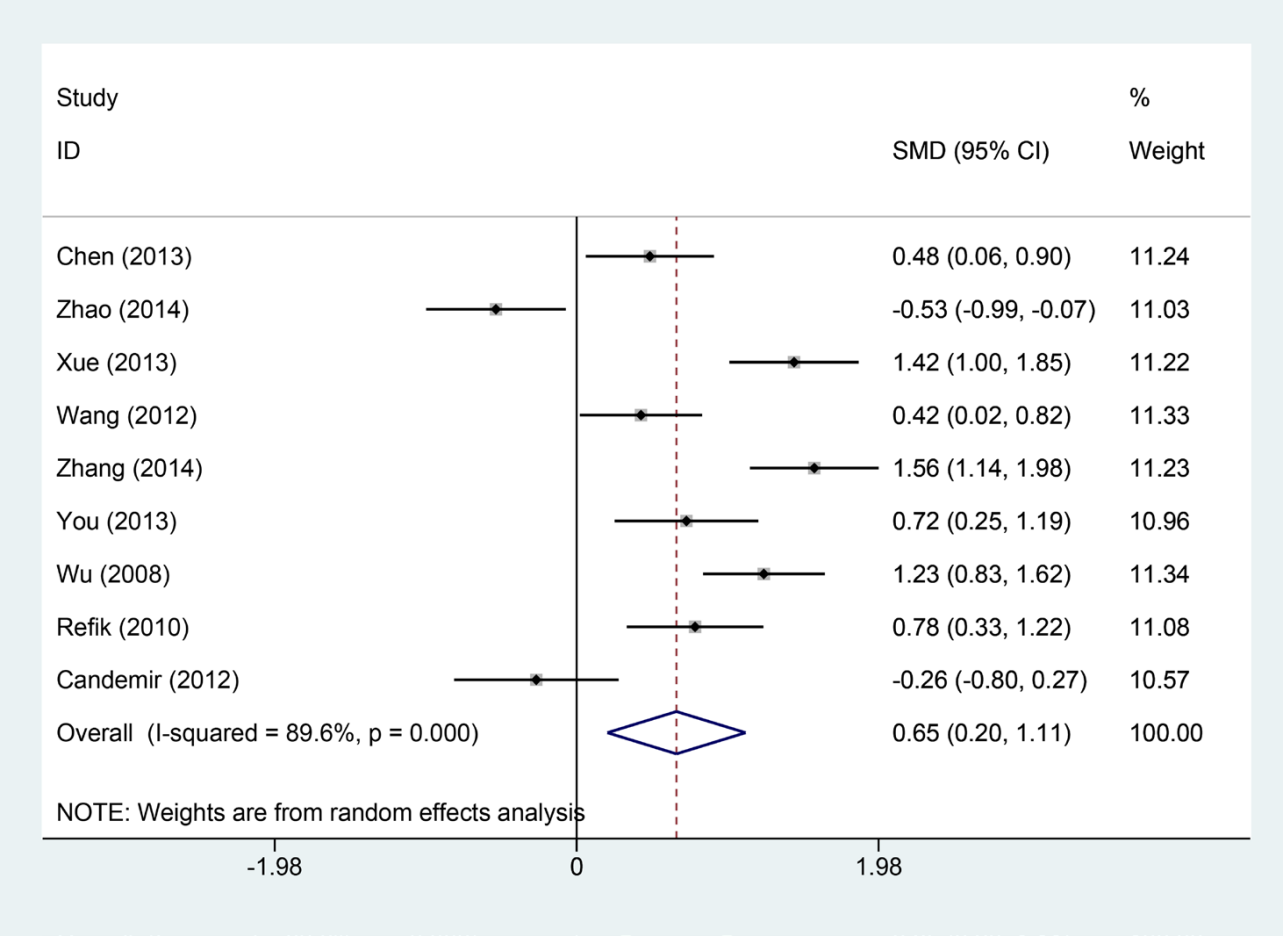
Comparisons of left ventricular ejection fraction between intracoronary vs intravenous

Thirsty studies totaling 1550 patients reported results on thrombolysis in myocardial infarction trial. The heterogeneity within studies was high (I^2^ = 85.9%, *P* = 0.000), and random-effect model was conducted. Compared with intravenous, intracoronary of Tirofiban could increase the incidence of TIMI III level (RR = 1.17, 95% CI: 1.11–1.22, Figure 2). Nine studies reported the left ventricular ejection fraction with a total number of 716 patients. The high heterogeneity was found within studies, and the random-effect was used (I^2^ = 89.6%, *P* = 0.000). Compared with intravenous, intracoronary of Tirofiban could increase the left ventricular ejection fraction (SMD = 0.65, 95% CI: 0.20–1.11, Figure 3).

**Figure 4:**
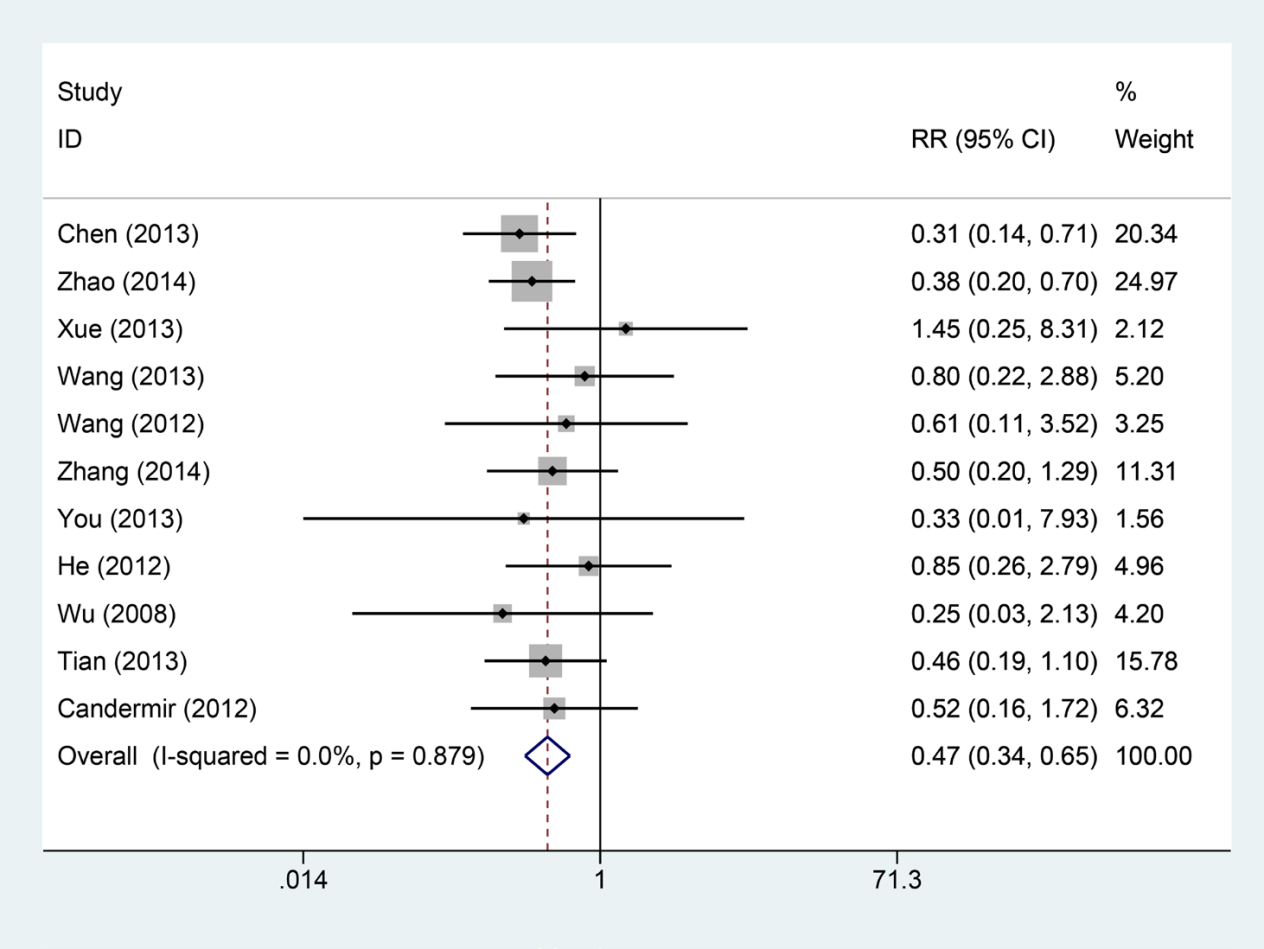
Comparisons of cardiovascular adverse events incidences between intracoronary vs intravenous

**Figure 5:**
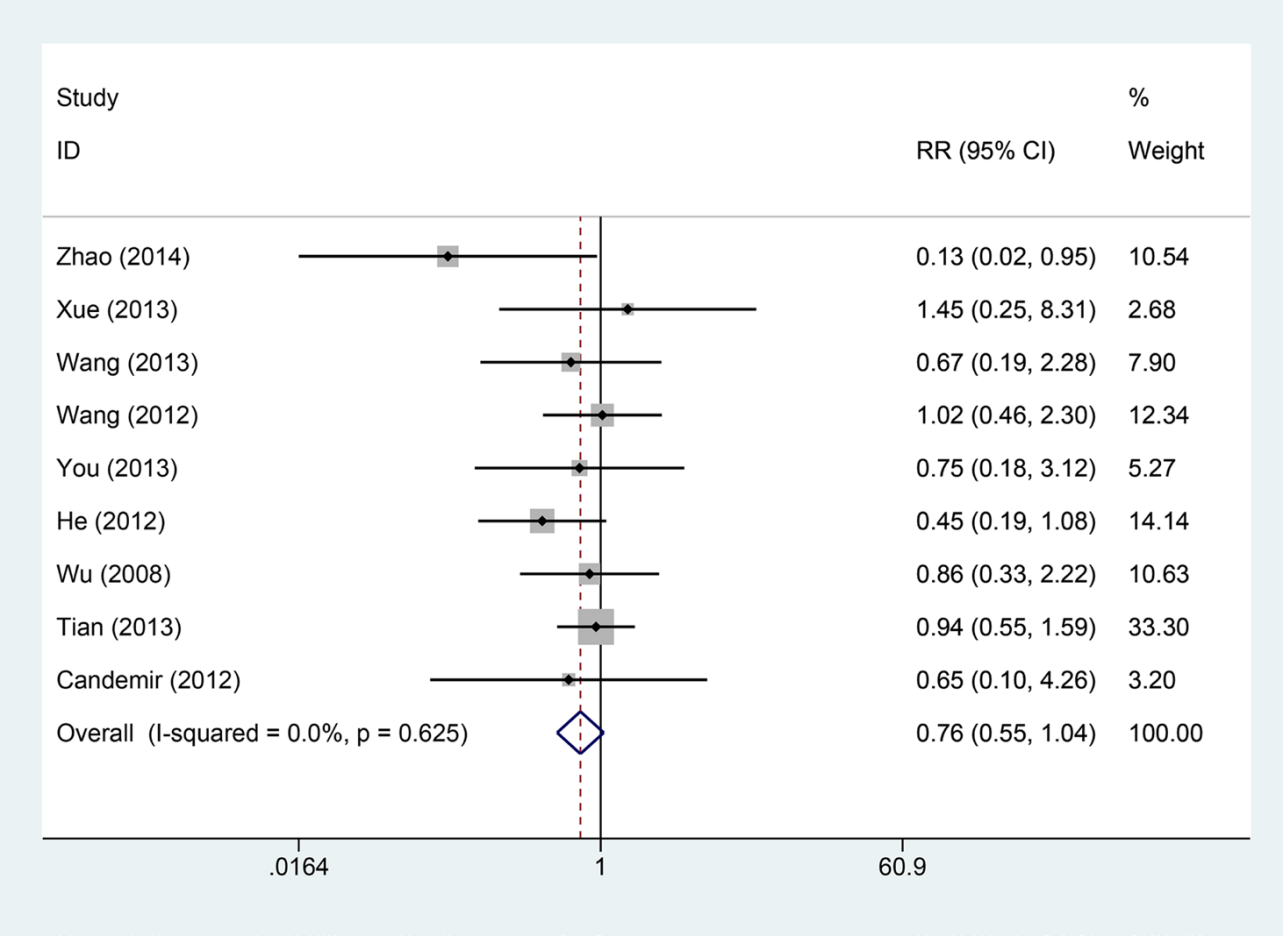
Comparisons of bleeding complication incidences between intracoronary vs intravenous

Eleven studies provided data on cardiovascular adverse events. The intracoronary Tirofiban reduced the risk of cardiovascular adverse events with low heterogeneity (I^2^ = 0.0%, *P* = 0.879, Figure 4). The relative risk and its 95% confidence interval was 0.47 (0.34–0.65). Nine studies reported the results of bleeding complications. The results from fixed-effect model indicated that there was no statistical significance between intravenous and intracoronary group (I^2^ = 0.0%, *P* = 0.625; RR = 0.76, 95% CI: 0.55–1.04, Figure 5).

#### Sensitivity analyses and publication bias

To examine the stability of pooled results, we conducted sensitivity analyses for each pooled result. The Supplementary Figure 2 (S2-A-D) presented the sensitivity analyses results. The results did not change greatly for ejection fraction (S2 B), cardiovascular adverse events (S2 C), and bleeding complications (S2 D). For thrombolysis in myocardial infarction trial, the results changed a lot when two studies were excluded [8, 18]. However, the whole trend of improved TIMI was not altered. These findings shown robust pooled results. We also calculated the power of the meta-analysis, the power of this meta-analysis ranged from 81% to 89%.

**Figure 6:**
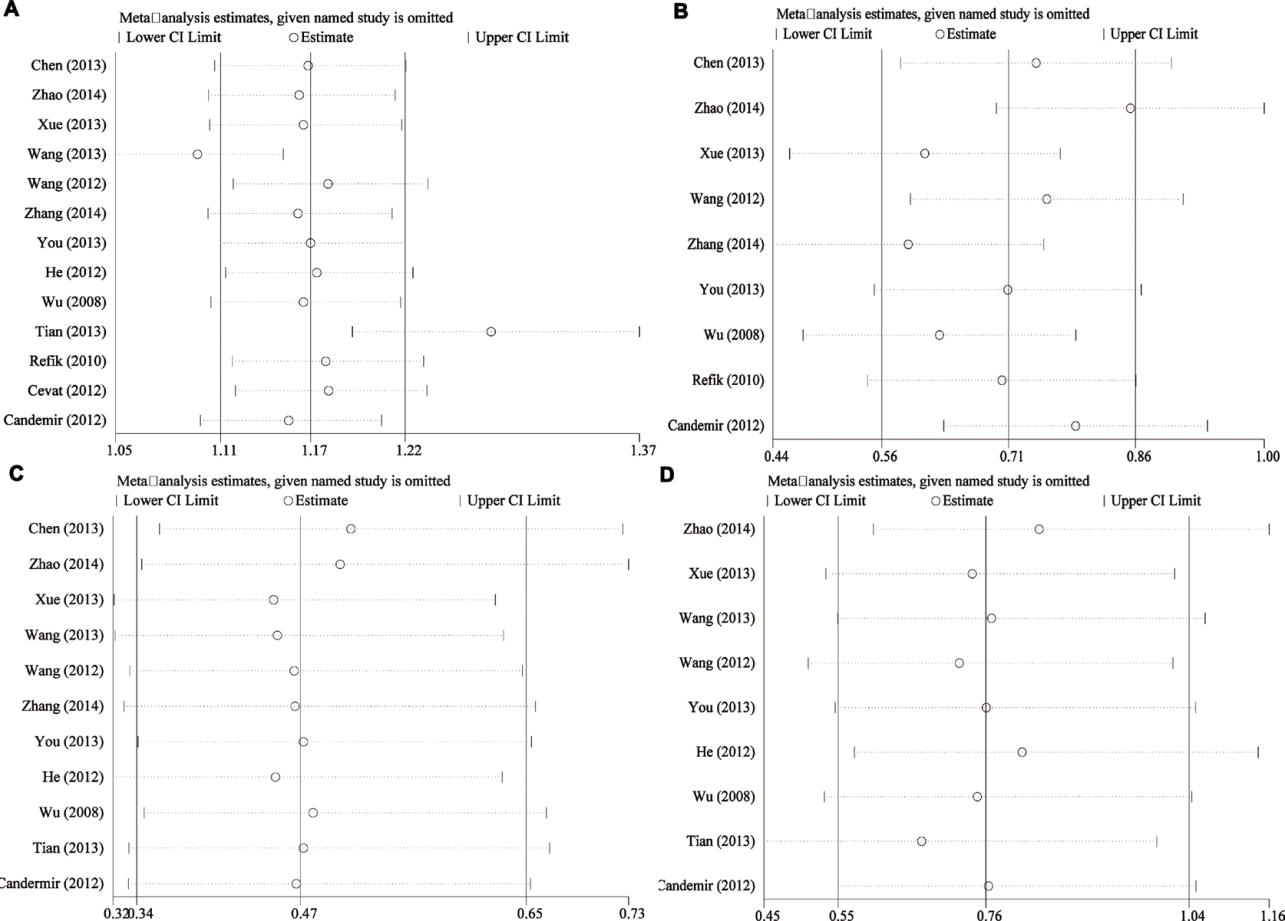
Funnel plot of publication bias

The funnel plot was used to evaluate the publication bias. There were slightly asymmetric via visual judgement (Figure 6). The Egger and Begger’ test also gave the same results (Table 2).

**Table 2:** Pooled results of comparison of intracoronary versus intravenous administration of tirofiban in patients with percutaneous coronary intervention

Outcomes	*N*	*I*^2^(%)	*P*_hetero_	Model	Effect value (95% CI)	Publication bias	
						Begg	Egger
TIMI	13	85.9	0.000	Random	1.17(1.11–1.22)	0.028	0.022
Left ventricular EF	9	86.9	0.000	Random	0.65(0.20–1.11)	0.297	0.126
Cardiovascular adverse events	11	0.0	0.897	Fixed	0.47(0.34–0.65)	0.139	0.151
Bleeding complications	9	0.0	0.625	Fixed	0.76(0.55–1.04)	0.139	0.151

*P*_hetero_*: P* values for heterogeneity; RR: relative risk; CI: confidence interval; TIMI: thrombolysis in myocardial infarction trial; EF: ejection fraction.

## DISCUSSION

Our study found that (1) patients with intracoronary administration tended to reach thrombolysis in myocardial infarction trial grade 3 flow after PCI; (2) patients of left ventricular ejection fraction were improved compared with intravenous administration; (3) patients with intracoronary administration resulted in a reduced risk of major adverse cardiovascular events by 47% after 30-day’s follow-up. (4) Patients’ incidence of bleeding in intracoronary group was almost equal to that of intravenous group. One meta-analysis on this topic had been published [19]. Thought some findings of our study was in accordance with previous one. Differences between ours and the previous one should be indicated. First, the previous study only included no more than seven articles with 1027 patients. In comparison, our study included 13 studies with a total number of 1550 patients. Our study was the latest with higher statistical power. Second, the estimation of complete perfusion consisted of five studies, and this was really under power. Finally, the previous one used funnel plot to evaluate the publication bias, and it was inappropriate for evaluation of publication bias when number of study was less than ten according to Cochrane handbook [20].

Tirofiban is an antiplatelet drug. It belongs to a class of antiplatelet named glycoprotein IIb/IIIa inhibitors with 2 hours’ biological half-life. Tirofiban is the first drug candidate whose origins can be traced to a pharmacophore-based virtual screening lead [21]. The fibrinogen was combined with activated platelets in the final stage of platelet aggregation. This process completely depended on regulation of GP IIb/IIIreceptor on platelet surface. The incidence of postoperative bleeding would reduce if the platelet was fully inhibited [22]. It took 10–30 minutes for Tirofiban to reach the peak of plasma concentrations by the way of intravenous administration, its efficiency would be down because of the first pass metabolism effect. Patients with acute coronary syndrome, especially for patients with ST-segment elevation myocardial infarction, usually did not have antegrade blood flow. It was difficult for Trirobifan to reach the lesions of coronary artery [23]. However, the intracoronary administration of Tirofiban avoided the above shortcomings, achieved high drug concentration in coronary artery and thrombosis. Besides, it accelerated the aggregation of platelets and inhibited the format of microthrombus, promoted myocardial perfusion, and stopped the progress of myocardial necrosis [24]. Intracoronary administration was a priority option for patients after PCI operative. GPIs had definite antiplatelet aggregative activity, and the primary adverse effect was bleeding complications and thrombogenic, which must be taken into consideration, especially in usage of aspirin and clopidogrel. However, recent studies reported that no significant differences in bleeding and thrombogenic were observed between intracoronary administration and intravenous. These results show the safety of intracoronary administration of Tirofiban was under control [25].

Several limitations of this meta-analysis merit consideration. The first limitation is the duration of follow-up. The evaluation of cardiovascular adverse events was within 30 days, and the long-term effect cannot be observed. Longer follow-up was required. Secondly, almost all the included studies did not use blinding method to study procedure, which could have resulted in performance and detection bias. Thirdly, the funnel plot indicated there were slightly asymmetric, which means publication bias may exist. This publication bias existed in the thrombolysis in myocardial infarction trial setting. This may be associated with different follow-up duration. But the subgroup analyses did not give the source of bias. Finally, some studies population in included studies were conducted in different settings. The potential risk of introducing significant heterogeneity was possible.

In conclusion, the present study found that intracoronary administration of Tirofiban can effectively increase the blood flow of coronary artery and microvascular perfusion, promoted functional recovery of left ventricular, and reduced the incidences of cardiovascular adverse events without the elevated risk of bleeding during 30-day’s follow-up.

## MATERIALS AND METHODS

### Search strategy

We conducted a comprehensive literature search in the electronic databases of PubMed, Web of Science, China National Knowledge Infrastructure, and Wangfang from inception to May 1st, 2017. We used the MeSH terms and free-text words to increase the accuracy and sensitivity of the search. The following key words were used in combinations: “Acute coronary syndrome”, “Percutaneous coronary”, “tirofiban”, randomized controlled trials. We placed restriction in English and Chinese. We also screened the references of retrieved relevant articles to identify potentially eligible literatures.

### Criteria for inclusion and exclusion

The included study had to meet the following criteria: (I) study design: randomized controlled trial design or cohort study; (II) study subject: patients who underwent percutaneous coronary intervention received Tirofiban intracoronary or intravenous administration; (III) intervention: trial group received intracoronary Tirofiban during PCI, and control group took intravenous Tirofiban during operation. The following patients were excluded: patients with cardiac insufficiency, liver or renal dysfunction, diabetes, history of myocardial infarction, sever infection, injury, malignant tumor, connective tissue disease, water and electrolyte disorder, blood disease, thyroid disease, chronic obstructive pulmonary disease, pulmonary embolism.

### Data extraction and assessment of quality

Two authors extracted the data independently, and disagreement were resolved by the third author. The following information was extracted: the first author, year of publication, duration of follow-up, sample size. The outcomes included the incidence of thrombolysis in myocardial infarction, left ventricular ejection fraction, occurrence of cardiovascular adverse events (mortality, angina pectoris, and arrhythmia), incidence of complications, and cardiogenic shock.

We used the bias of risk tools recommended by Cochrane Collaboration to assess the quality of included studies [26]. This tool consisted of random sequence generation, allocation concealment, blinding of participants and personnel to the study protocol, blinding of outcome, incomplete data and selective reporting and other bias. We scored low, high and unclear risk of bias according to the criteria above. Implementation of blinding and concealment was usually extremely difficult for clinical treatment trials.

### Statistical analysis

Relative risks and 95% confidence intervals were calculated to assess the efficacy and safety of tirofiban intracoronary versus intravenous administration during percutaneous coronary intervention for patients with acute coronary syndrome. Statistical heterogeneity across studies was assessed by a standard chi-square test and I^2^ statistics. I^2^ > 50% or *P* < 0.05 was considered to indicate substantial heterogeneity [27]. The random-effect model was used for existed heterogeneity, or fixed-effect model was used. Sensitivity analyses were also conducted to test the stability of pooled results. Publication bias was assessed by visually inspecting a funnel plot and evaluated using the Begg and Egger’s test [28, 29]. All analyses were performed using Stata 14.0 (Stata Corp. LP) and RevMan 5.3. *P* < 0.05 was considered as significance.

## SUPPLEMENTARY MATERIALS FIGURES



## Abbreviations

PCI: Percutaneous coronary intervention
TIMI: Thrombolysis in myocardial infarction trial
RR: Relative risk
CI: Confidence interval
SMD: Standardized mean difference
