# Supplementary material for: Intracoronary administration of tirofiban during percutaneous coronary intervention facilitates patients with acute coronary syndrome
Source: Oncotarget. 2017 Jul 12;8(63):107303–11. doi: 10.18632/oncotarget.19179 (PMC5739815; doi:10.18632/oncotarget.19179)
Supplement: Supplementary file 1 [file oncotarget-08-107303-s001.pdf]

Intracoronary administration of tirofiban during percutaneous coronary intervention facilitates patients with acute coronary syndrome

SUPPLEMENTARY MATERIALS

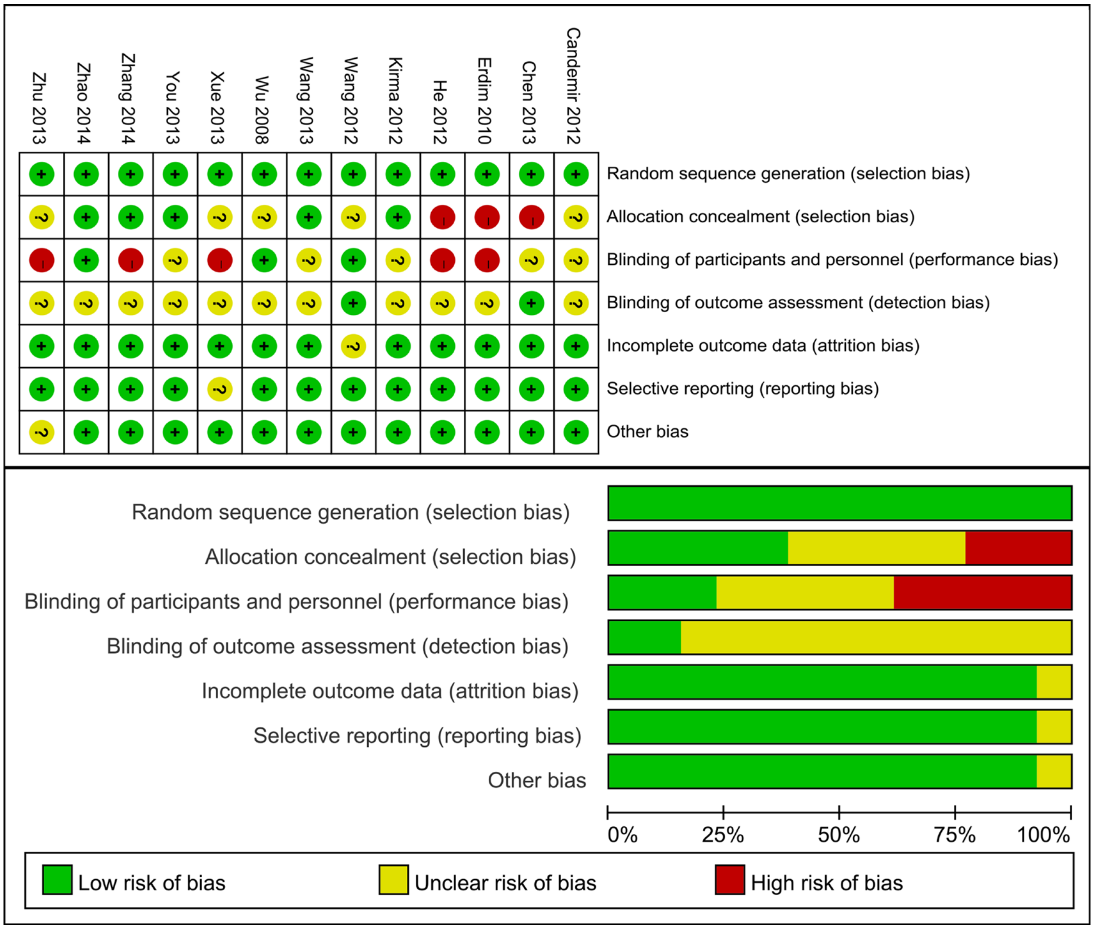

Supplementary Figure 1: Assessment of quality for included studies.

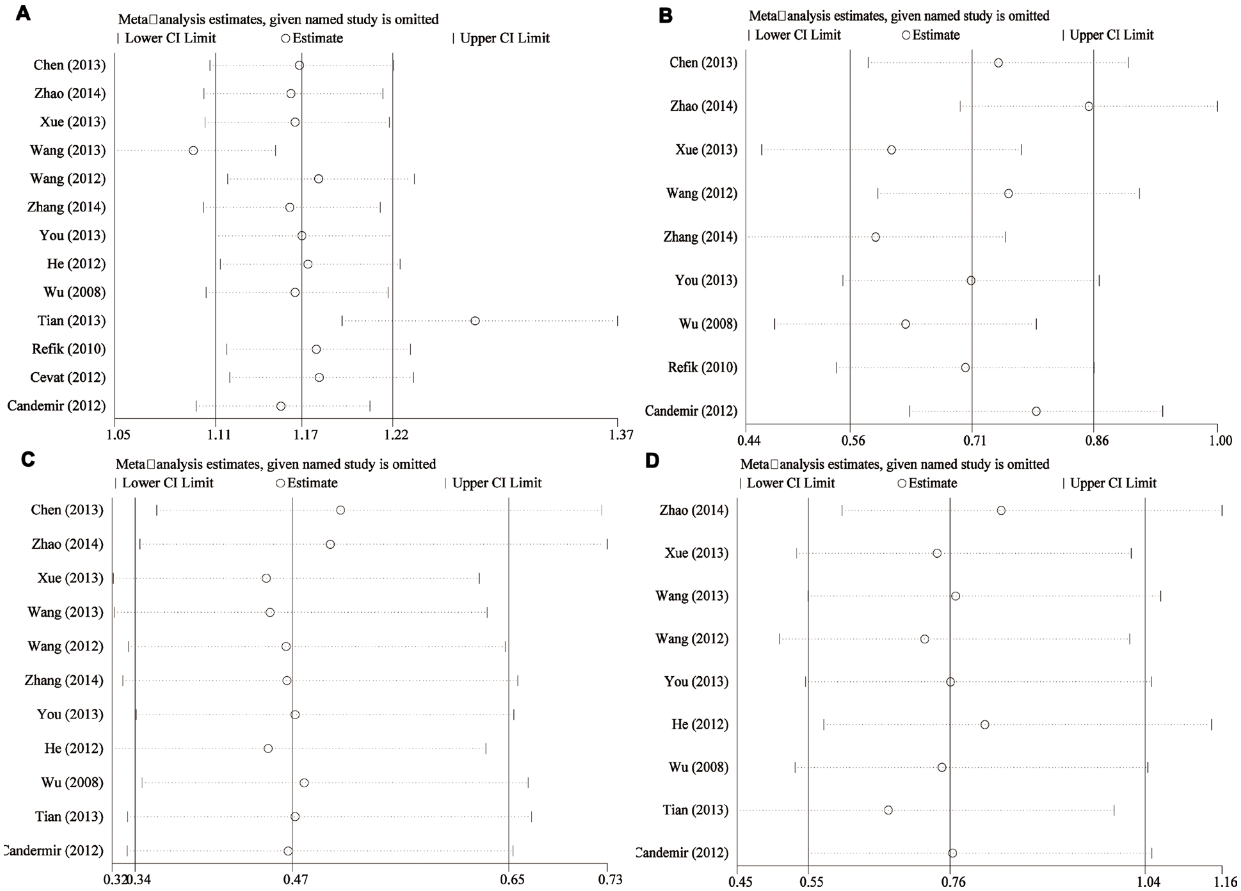

Supplementary Figure 2: Sensitivity analyses of pooled results (**A**) thrombolysis in myocardial infarction, (**B**) ejection fraction, (**C**) cardiovascular adverse events, (**D**) bleeding complications).
